# We See That Plastics Are Persistent. But Should We Treat Them Universally as a POP?

**DOI:** 10.1021/acs.est.5c18400

**Published:** 2026-03-25

**Authors:** Melissa A. Maurer-Jones, Naba K. Kalita

**Affiliations:** † Department of Chemistry and Biochemistry, 14713 University of Minnesota Duluth, 1038 University Drive, Duluth, Minnesota 55812, United States; ‡ Natural Resource Research Institute, University of Minnesota, 5013 Miller Trunk Highway, Duluth, Minnesota 55811, United States

**Keywords:** micro- and nanoplastics, persistent organic pollutants, environmental lifetimes, reactivity

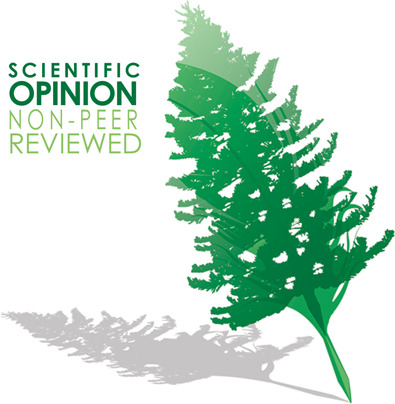

## Graphical abstract

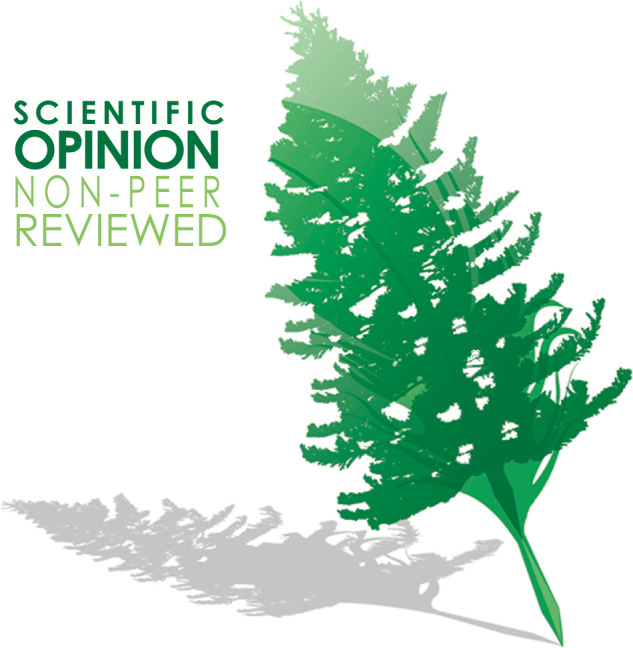

Plastics in the environment, both as bulk and micro- and nanoplastics, has been a growing area of research for the past decades, with an exponential increase in the field in the past 10 years. There is a consensus that some polymer types could be considered persistent in that they are detectable in many locations, even remote areas with minimal human activity. There are debates on the half-life of these materials in various environmental compartments, and it is becoming increasingly clear that original reports of plastic lifetime may not be as long-lived as originally reported.^1^ With that said, a growing body of evidence of the bioaccumulative and toxic potential of plastics and/or the chemicals that can leach raises the question: should we universally consider all plastics to be persistent organic pollutants (POPs)?

## POPs Have Long Environmental Lifetimes

The first characteristic of POPs is longevity in the environment. While many polymers have been thought to be in the environment for decades and even centuries, defining the lifetime of a plastic in our natural systems depends on both the polymer material and the environmental conditions (e.g., surface water or sediment). Beyond system variability, studies often approach the calculation of degradation rates using different metrics or measurements, which makes it challenging to incorporate environmental degradation into modeling of persistence or life cycle assessments. Recent efforts have worked to harmonize degradation rates of legacy, petroleum-derived polymer materials (i.e., commodity plastics) using specific surface degradation rates^2^ that result in values of mass or size loss over time (e.g., micrometers per year). However, metrics based on size reduction alone will fail to distinguish between abiotic degradation and complete biodegradation. To determine if a plastic is truly “persistent” in the context of POPs, researchers have been encouraged to distinguish between abiotic disintegration and biodegradation through respiratory metrics, such as conversion to biomass or CO_2_/CH_4_.^3^

Applying degradation rates is crucial as we move forward and aim to use plastic materials sustainably,^4,5^ but it raises a fundamental question of whether biodegradable and compostable plastics should be grouped with conventional commodity plastics. Although this is a step forward, the lifetime estimates are enormously wide, which limits the predictive power of our persistence assessments and may miss some plastic-specific context that will limit its applicability.

## Lifetimes Are Related to Reactivity

Given that our understanding of lifetimes is still improving for plastics, establishing reactivity metrics such as those for POPs could deepen our understanding of persistence. Modeling structure–activity relationships based on chemical properties has been an important tool to predict the reactivity and persistence of organic chemicals. These approaches are important because they apply observational or experimental data with *in silico* analysis to determine the fate, transformations, and impact of these compounds in the environment.^6^ For plastics, these analyses have the potential to take into account how the chemical structure could impact the polymer properties. In one example, the U.S. Department of Energy National Laboratory of the Rockies has developed PolyID that predicts the thermal and mechanical properties of a polymer based on its chemical structure.^7^ Unfortunately, this tool does not take into account the behavior of the polymer if there are random chemical changes within the backbone of the polymer as a result of weathering (e.g., photo-oxidation causing oxygen moieties on polyethylene backbone), nor does it take into account the fact that material properties, and thus degradation pathways, can be impacted by characteristics beyond the chemical structure of the polymer. In other words, plastic material properties vary with the addition of additives, pigments, and processing conditions such as temperature that ultimately influences its environmental transformations and fate.

The major challenge with applying these tools for predicting degradation and ultimately persistence is that most commercial plastics themselves are a mixture (polymer and additives) in a complex and dynamic natural system. The field of engineered nanoparticles in the environment has faced a similar problem. Surface reactivity is an important characteristic of engineered nanoparticles and often imparts their physical and optical properties. Some work has sought to characterize the nanoparticle surface reactivity with natural systems,^8^ but primary modeling and prediction of nanoparticle fate and effects have been with quantitative structure–activity or −toxicity relationships (QSAR or QSTR, respectively).^9^ Quantitative structure activity–property relationships, popular tools for statistical and computational modeling of POPs, have been applied to the fate of plastics, but they have primarily been associated with predicting the adsorption of contaminants to the surface of plastics.^10^ Continued development of QSAR-type models for plastics is urgently needed to properly assess reactivity.

If reactivity, particularly surface reactivity, is critical for predicting the fate, transformation, and persistence of polymeric materials, then acknowledging the “eco-corona” is crucial. In other words, plastics accumulate molecules and organisms on their surface as it moves through the environment and these play an important role in its degradation pathways. There is already evidence that attached biofilms will slow photochemical breakdown of polymeric materials,^11^ while simultaneously, it also creates a microenvironment, the “plastisphere,”^12^ that may accelerate biodegradation.^13^ This duality creates a perfect “persistence paradox,” a complexity current models must capture in our prediction of persistence.

## Should We Classify Micro- and Nanoplastics as POPs?

The argument to classify micro- and nanoplastics as POPs may be important from a policy perspective.^14^ Beyond the growing body of evidence of adverse biological effects, the modeling community suggests that the property of persistence in itself is a critical hazard criterion.^15^ In other words, while chemicals have diverse chemical and physical properties, evidence suggests that simply due to their persistence, there will be increased probabilities of known and unknown effects due to continued release and that it would take decades or even centuries to reverse these effects. Seeing through the lens of socio-economic dimensions, such as the “essential use” concept, and when applied on persistent plastics, it shifts the evaluation to socio-economic functionality beyond its chemical functionality.^16^ Consequently, societal constraints drive materials to be judged by their current waste management infrastructure, rather than by theoretical properties alone. This socio-economic viewpoint moves governance from theoretical material properties to practical and risk-based rationality^17,18^ and could suggest plastics in micro- or nanosized forms be categorized as POPs.

However, based on current definitions of POPS and a chemical standpoint, micro- and nanoplastics do not fit the regulatory criterion. There is a growing body of evidence that the material behavior in the environment is as diverse as the types of plastic products, and we would argue that our understanding of persistence and reactivity of these materials is evolving. In addition, the emergence of new polymer types that have environmental degradation integrated within their design (i.e., biodegradable and biobased plastics) may aid in our dependency on commodity plastic and have significantly decreased lifetimes. We agree that plastics and their prevalence are a significant problem; however, there are many questions remaining around their persistence, and the blunt instrument of the POP label for all plastic waste loses the scientific accuracy needed to capture the full scope of the problem. While logistically complicated, regulatory actions on plastics should be considered based on the hazards posed by specific plastic compositions.
